# Comparison of the Effects of Transarterial Chemoembolization for Advanced Hepatocellular Carcinoma between Patients with and without Extrahepatic Metastases

**DOI:** 10.1371/journal.pone.0113926

**Published:** 2014-11-26

**Authors:** Jeong-Ju Yoo, Jeong-Hoon Lee, Sang Hwan Lee, Minjong Lee, Dong Hyeon Lee, Yuri Cho, Yun Bin Lee, Su Jong Yu, Hyo-Cheol Kim, Yoon Jun Kim, Jung-Hwan Yoon, Chung Yong Kim, Hyo-Suk Lee

**Affiliations:** 1 Department of Internal Medicine and Liver Research Institute, Seoul National University College of Medicine, Seoul National University Hospital, Seoul, Republic of Korea; 2 Department of Radiology, Seoul National University College of Medicine, and Institute of Radiation Medicine, Seoul National University Medical Research Center, Seoul, Korea; Yonsei University College of Medicine, Republic of Korea

## Abstract

**Background/Aims:**

Sorafenib is a standard treatment for advanced hepatocellular carcinoma (HCC) (Barcelona Clinic Liver Cancer [BCLC] stage C). However, transarterial chemoembolization (TACE) has also been widely used as a treatment for patients with advanced HCC, even if they have extrahepatic metastases (EHM). The aim of this study was to determine the efficacy of TACE for advanced HCC patients with EHM upon initial diagnosis, as compared with those patients without EHM.

**Methods:**

This cohort study involved consecutive patients who underwent TACE as an initial treatment for advanced HCC. One hundred seventy-seven patients with EHM (the EHM group) and 205 with portal vein invasion without EHM (the non-EHM group) were included. A survival analysis was performed to compare overall survival between the two groups.

**Results:**

The mean age was 54.5±9.9 years, and median follow-up duration was 13.1 months (range, 0.5–111.0). Overall survival was significantly shorter in the EHM group than the non-EHM group (median, 8.3 vs. 19.1 months; *P*<0.001). A multivariate analysis showed that the presence of EHM was an independent poor prognostic factor for shorter overall survival (adjusted hazard ratio, 1.74; 95% confidence interval, 1.39–2.17; *P*<0.001) after adjustment for Child-Pugh classification, intrahepatic tumor T classification, tumor response to TACE, and serum alpha-fetoprotein level. Patients administered TACE and systemic therapy demonstrated a better survival rate than those administered TACE alone in both the EHM (median, 13.5 vs. 7.2 months) and non-EHM groups (median, 27.9 vs. 18.2 months) (both, *P*<0.05).

**Conclusions:**

The prognosis of advanced HCC patients with EHM is significantly worse than those without EHM administered repeated TACE treatments, even if their tumor stage was similar to BCLC stage C. These results suggest that EHM presence means aggressive tumor biology and that BCLC stage C might be subclassified according to EHM presence.

## Introduction

In the past, the presence of extrahepatic metastases (EHM) upon initial diagnosis of hepatocellular carcinoma (HCC) was relatively rare, even with fairly advanced intrahepatic lesions [1]. Recently, however, EHM associated with HCC has been increasing because of increased survival following effective loco-regional therapies [2]. Moreover, improvements in diagnostic techniques such as ultrasonography (US), computed tomography (CT), magnetic resonance imaging (MRI) and positron emission tomography (PET) with ^18^F-fluorodeoxyglucose have led to better detection of EHM in patients with HCC [3].

At present, the prognosis of patients with EHM from primary HCC is poor [4]. In this regard, there is little information regarding the cause of death for these patients, and there is no consensus regarding the treatment strategy for EHM associated with HCC. According to the Barcelona Clinic Liver Cancer (BCLC) staging system, sorafenib is recommended for HCC patients who have advanced stage (stage C) disease, with or without EHM. However, sorafenib demonstrated only a modest improvement over that of placebo [5], [6].

Previous reports suggest that the cause of death for HCC patients with EHM may primarily be because of intrahepatic HCC, hepatic failure secondary to progression of intrahepatic HCC, or underlying liver disease, rather than EHM [2], [4]. Although the metastatic disease might not be cured, a loco-regional treatment modality such as transarterial chemoembolization (TACE), rather than systemic treatment, could offer some survival benefits [1].

Until now, data regarding the efficacy of TACE for the survival of metastatic HCC patients are scarce. The aim of this present study was to: (i) compare the overall survival (OS) of advanced HCC patients with EHM, as compared with those who did not have EHM and who were administered TACE as an initial therapy and (ii) to evaluate the factors affecting overall survival in advanced HCC.

## Materials and Methods

### Patients

Written consent was not obtained, because the participants were to remain anonymous and the data were analyzed anonymously. The study protocol was approved by the Institutional Review Board of Seoul National University Hospital. The study protocol conformed to the ethical guidelines of the World Medical Association Declaration of Helsinki and was approved by the Institutional Review Board of Seoul National University Hospital (IRB No. H-1409-025-607).

A consecutive series of 1,313 HCC patients who underwent TACE as an initial treatment were investigated at a tertiary hospital (Seoul National University Hospital; Seoul, Korea) between January 2005 and December 2008. We retrospectively enrolled 382 patients who had BCLC stage C disease at the time of their initial diagnosis. Those BCLC stage C patients were divided into two groups: patients with EHM (the EHM group) and patients with portal vein invasion without EHM (the non-EHM group). Patients were excluded if they had (a) poor hepatic function classified as Child-Pugh class C or poor performance status (BCLC stage D), (b) previous or current malignancy except for HCC, or (c) other loco-regional or systemic therapy prior to TACE.

### Diagnosis and procedure

Diagnosis and staging of HCC were based on the American Association for the Study of Liver Diseases criteria and BCLC and American Joint Committee on Cancer staging system 7th edition (AJCC-7) staging systems [7]–[9]. To screen for extrahepatic metastasis, patients routinely underwent a standard history-taking and physical examination. Palpable lymph node was evaluated during physical examination at the time of initial diagnosis. Intra-abdominal metastatic lesion (e.g. lymph node metastasis, adrenal gland metastasis, peritoneal implantation, etc.) was evaluated using dynamic CT or MRI covering abdomen and pelvis. To evaluate lung metastasis, plain chest X-ray was routinely performed. If suspicious lung lesion was found on either chest X- ray or abdominal CT covering lower part of the both lung fields, additional chest CT or whole body PET was performed. Studies such as bone scans and brain MRI were performed when patients have symptoms or signs suggesting the presence of metastasis to regarding organs, such as bone pain, neurological abnormality, and so on. These examinations were also conducted when the serum level of AFP or protein-induced by vitamin K absence-II was elevated and the elevations could not be attributed to the status of the intrahepatic lesions. EHM was diagnosed using CT, MRI, chest X-ray, bone scans, PET with ^18^F-fluorodeoxyglucose, or any combination of these. If metastases occurred at multiple sites, each metastasis was separately counted for each location.

TACE was performed using a previously described method [10], [11]. If possible, superselective chemoembolization was performed through the lobar, segmental, or subsegmental arteries, depending on tumor distribution and hepatic function reserve. The extent of chemoembolization was individually adjusted using a superselective catheterization technique. The procedure was initially performed by infusing 2–12 mL of iodized oil (Lipiodol; Andre Gurbet, Aulnay-sous-Bois, France) and 10–60 mg of doxorubicin hydrochloride emulsion (Adriamycin RDF; Ildong Pharmaceutical, Seoul, Korea) until arterial flow stasis was achieved and/or iodized oil appeared in the portal branches. The doses of doxorubicin and iodized oil were individually determined according to tumor size, and the liver function. The total amount of cisplatin used for treatment was 50 mg/m2 body surface area and it was infused into the tumor-feeding vessels at a rate of 4–10 mL/min. Contrast-enhanced dynamic CT was performed 4 weeks after TACE to assess the need of a consecutive treatment. Repeated TACE was performed every 6–8 weeks if any viable tumors were detected on sequential imaging, even if the treatment response of previous TACE is PR or SD, without deterioration of liver function.

### Image analysis

After the initial TACE treatment, tumor response was evaluated according to Modified Response Evaluation Criteria in Solid Tumors (mRECIST) [12]. Two radiologists (S.H.L. and H.C.K.) with over 5 years' experience were involved in the analysis. Arterial phase CT or MRI was used for response assessment. Tumor response to TACE was defined as: (i) complete response (CR, the disappearance of intratumoral arterial enhancement in all target lesions); (ii) partial response (PR, at least a 30% decrease in the sum of viable target lesion diameters); (iii) progressive disease (PD, at least a 20% increase in the sum of enhanced target lesion diameters); and (iv) stable disease (SD, any case that did not qualify as either partial response or progressive disease). Patients with CR or PR were defined as responders, whereas patients with SD or PD were defined as non-responders.

### Data collection and primary end point

Clinical, laboratory, and radiologic records of all patients were retrospectively reviewed. To determine differences in the therapeutic efficacy of TACE between the two groups, we compared survival rates and assessed OS as a primary endpoint. OS was defined as the time from the date of HCC diagnosis until the date of death from any cause. The cause of death was evaluated, if possible.

### Statistical analysis

Frequencies and percentages were used for descriptive statistics. Statistical differences between the two groups were investigated using the χ^2^ test and Student's *t* test. Patient survival probability was estimated using the Kaplan-Meier method, and differences between the curves were compared using the log-rank test. The main analysis tool used for survival was the Cox proportional hazards model. Multivariate models were created using variables that were significant in a univariate analysis (*P*<0.10) and clinically relevant. All statistical analyses were performed using PASW version 18.0 (SPSS Inc; Chicago, IL), and statistical significance was defined as a *P* of <0.05.

## Results

### Baseline characteristics and additional treatment

Among the 382 patients with BCLC stage C disease who were reviewed, 177 had EHM with or without portal vein invasion (the EHM group), and 205 had portal vein invasion without EHM (the non-EHM group) at the time of their initial diagnosis. Patient characteristics are shown in Table 1. Their overall mean age was 54.5±9.9 years, and 88.5% were male. According to AJCC-7 system, 1 patient (0.3%) had intrahepatic HCC classified as T2; 343 patients (89.8%) were classified as T3; and 38 patients (9.9%) were classified as T4. Approximately 70% of the patients had Child-Pugh class A liver function. For the EHM group, the EHM involved the lung (n = 85), lymph node (n = 75), bone (n = 25), adrenal gland (n = 16), peritoneum (n = 19), and brain (n = 3), as well as other sites (n = 9). The rate of hepatitis B surface antigen (HBsAg) or anti-HCV positivity was not different between the two groups.

**Table 1:** Baseline characteristics of patients who have advanced HCC with and without extrahepatic metastases.

	All (n = 382)	EHM (n = 177)	Non-EHM (n = 205)	*P*
**Age (years) - mean ±SD**	54.5±9.9	55.3±9.9	53.8±9.9	0.117
**Male sex - no. (%)**	338 (88.5)	155 (87.6)	183 (89.3)	0.604
**AJCC T classification - no. (%)**				0.098
T2	1 (0.3)	1 (0.6)	0 (0)	
T3	343 (89.8)	153 (86.4)	190 (92.7)	
T4	38 (9.9)	23 (13.0)	15 (7.3)	
**Child-Pugh classification - no. (%)**				0.909
Class A - no. (%)	273 (71.5)	127 (71.8)	146 (71.2)	
Class B - no. (%)	109 (28.5)	50 (28.2)	59 (28.8)	
**HBsAg/anti-HCV - no. (%)**				0.480
+/−	314 (82.2)	137 (77.4)	177 (86.3)	
−/+	18 (4.7)	8 (4.5)	10 (4.9)	
+/+	5 (1.3)	4 (2.3)	1 (0.5)	
−/−	45 (11.8)	28 (15.8)	17 (8.3)	
**Site of extrahepatic metastasis - no. (%)**				
Lung		85 (48.0)		
Lymph node		75 (42.4)		
Bone		25 (14.1)		
Adrenal gland		16 (9.0)		
Peritoneum		19 (10.7)		
Brain		3 (1.7)		
Others		9 (5.1)		
**Number of TACE procedure - mean ±SD**	3.3±3.2	2.4±2.4	3.8±3.3	<0.001

Abbreviations: HCC, hepatocellular carcinoma; AJCC, American Joint Committee on Cancer; Anti-HCV Ab, anti-hepatitis C virus antibody; EHM, extrahepatic metastases; HBsAg, hepatitis B surface antigen; TACE, transarterial chemoembolization.

Repeated superselective TACE was performed every 3 months if residual or recurrent intrahepatic HCC existed and if the liver function was preserved with a Child score of ≤9. The median number of TACE procedures was two in the EHM group (range, 1–12) and three in the non-EHM group (range, 1–16).

Generally, the EHM was treated with systemic therapy, surgical metastasectomy, or radiation therapy if (i) patients were symptomatic because of EHM; (ii) tumors were located at critical sites (e.g., the brain or weight-bearing bones such as the spine or femur); or (iii) no residual intrahepatic HCC existed, although the EHM developed no symptoms. In the EHM group, 31 patients (17.5%) underwent concurrent systemic therapy: 9 were administered sorafenib as a first-line systemic therapy with/without following second-line cytotoxic chemotherapy, and 22 were administered cytotoxic chemotherapy with 5-fluorouracil (5-FU)-based regimens (e.g., 5-FU/cisplatin, capecitabine/cisplatin, or 5-FU/folinic acid/oxaliplatin) and/or local treatments (e.g. RT, metastatectomy) for EHM. The median duration of systemic therapy was 78 days (range, 2–600 days). Thirty-five patients (19.8%) underwent palliative radiotherapy for EHM: 33 underwent radiation therapy for extrahepatic lesions, while 2 patients were administered radiation therapy for both intra- and extrahepatic lesions. Nine patients underwent surgical metastatectomy. In comparison, the non-EHM group was administered less systemic therapy or radiation therapy. Only 17 (8.3%) underwent concurrent systemic therapy.

### Treatment response to respective treatment

Tumor response to initial TACE was evaluated according to mRECIST criteria for 350 patients (Table 2). No patient in either group was classified as CR. The proportion of non-responders (SD+PD) was higher in the EHM group than in the non-EHM group (35.8% vs. 20.2%; *P*<0.001).

**Table 2:** Treatment responses after initial TACE for patients with and without extrahepatic metastases.

mRECIST- no. (%)	EHM	Non-EHM	*P*
CR	0 (0)	0 (0)	
PR	104 (58.8)	150 (73.2)	0.005
SD	49 (27.7)	33 (16.1)	<0.001
PD	9 (5.1)	5 (2.4)	0.005

Abbreviations: TACE, transarterial chemoembolization; mRECIST, modified Response Evaluation Criteria In Solid Tumors; CR, complete response; PR, partial response; SD, stable disease; PD, progressive disease.

### Treatment response of extrahepatic metastasis to multidisciplinary therapies

We also evaluated treatment response for extrahepatic lesion. A total of 64 patients who was treated to EHM were evaluated (5 sorafenib only, 4 sorafenib followed by second-line cytotoxic chemotherapy, 11 cytotoxic chemotherapy only, 3 radiation therapy + cytotoxic chemotherapy, 4 cytotoxic chemotherapy + surgical metastasecteomy, 5 surgical metastasecteomy only, and 32 radiation therapy only). Of the 64 patients, 4 patients showed PR, 7 SD, and 46 PD. Seven patients could not assess treatment response because there were no follow-up images. In sorafenib, all the 9 patients showed PD. In cytotoxic chemotherapy, all the 22 patients showed PD. In surgical metastasectomy, 1 patient showed PR, 7 PD. In radiation therapy, 3 patients showed PR, 7 SD, and 21 PD.

### Overall survival

**Figure 1:**
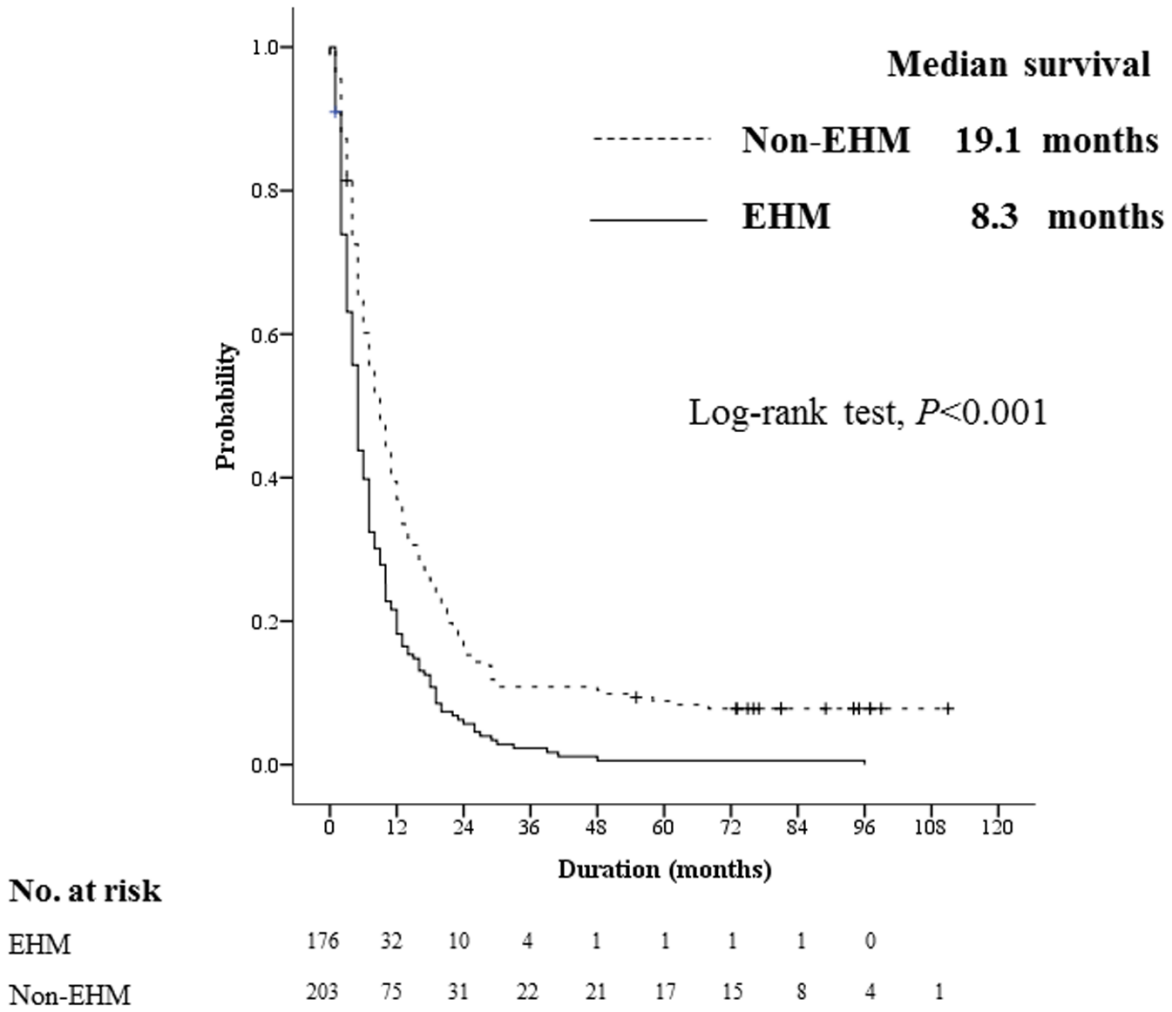
Kaplan-Meier survival curve for the EHM and the non-EHM group. Within the same BCLC stage C, the OS of the EHM group (solid line) was worse than the non-EHM group (dotted line) (median, 8.3 months vs. 19.1 months; *P*<0.001).

The median follow-up duration was 13.1 months (range, 0.5–111.0): 8.2 months (range, 0.5–96.0) in the EHM group and 17.4 months (range, 0.5–111.0) in the non-EHM group. During the study period, 176 patients in the EHM group and 187 in the non-EHM group died. The median OS was 8.3 months (range, 0.5–96.0) in the EHM group and 19.1 months (range, 0.7–111.0) in the non-EHM group. The mortality rate in the EHM group was approximately 1.8 times higher than that of the non-EHM group that underwent TACE (HR, 1.72; 95% CI, 1.40–2.12; *P*<0.001 by log-rank test; Figure 1). A multivariate analysis showed that the presence of EHM was an independent predictor of short OS (adjusted HR, 1.74; 95% CI, 1.39–2.17; *P*<0.001) after adjustment for Child-Pugh classification, AJCC T classification, tumor response (according to mRECIST), and serum alpha-fetoprotein level (Table 3).

**Table 3:** Univariate and multivariate analyses of predictive factors for overall survival.

Variables	Univariate analysis		Multivariate analysis	
	HR (95% CI)	*P*	HR (95% CI)	*P*
**Age (years)**				
0–60	1			
>60	0.80 (0.64–1.02)	0.66		
**Sex**				
Female	1			
Male	1.00 (0.72–1.39)	0.99		
**EHM**				
No	1		1	
Yes	1.72 (1.40–2.12)	<0.001	1.74 (1.39–2.17)	<0.001
**Child-Pugh classification**				
Class A	1		1	
Class B	1.78 (1.41–2.24)	<0.001	1.74 (1.35–2.24)	<0.001
**AJCC T classification**				
T3	1		1	
T4	1.78 (1.26–4.50)	<0.001	1.66 (1.13–2.43)	0.010
**mRECIST after 1st TACE**				
CR+PR	1		1	
SD+PD	1.84 (1.45–2.35)	<0.001	1.64 (1.28–2.11)	<0.001
**AFP (ng/mL)**				
0–200	1		1	
>200	1.53 (1.23–1.91)	<0.001	1.47 (1.16–1.84)	0.001

Abbreviations: EHM, extrahepatic metastases; AFP, serum α-fetoprotein; AJCC, American Joint Committee on Cancer; mRECIST, modified Response Evaluation Criteria In Solid Tumors; CR, complete response; PR, partial response; SD, stable disease; PD, progressive disease; HR, hazard ratio; CI, confidence interval.

**Figure 2:**
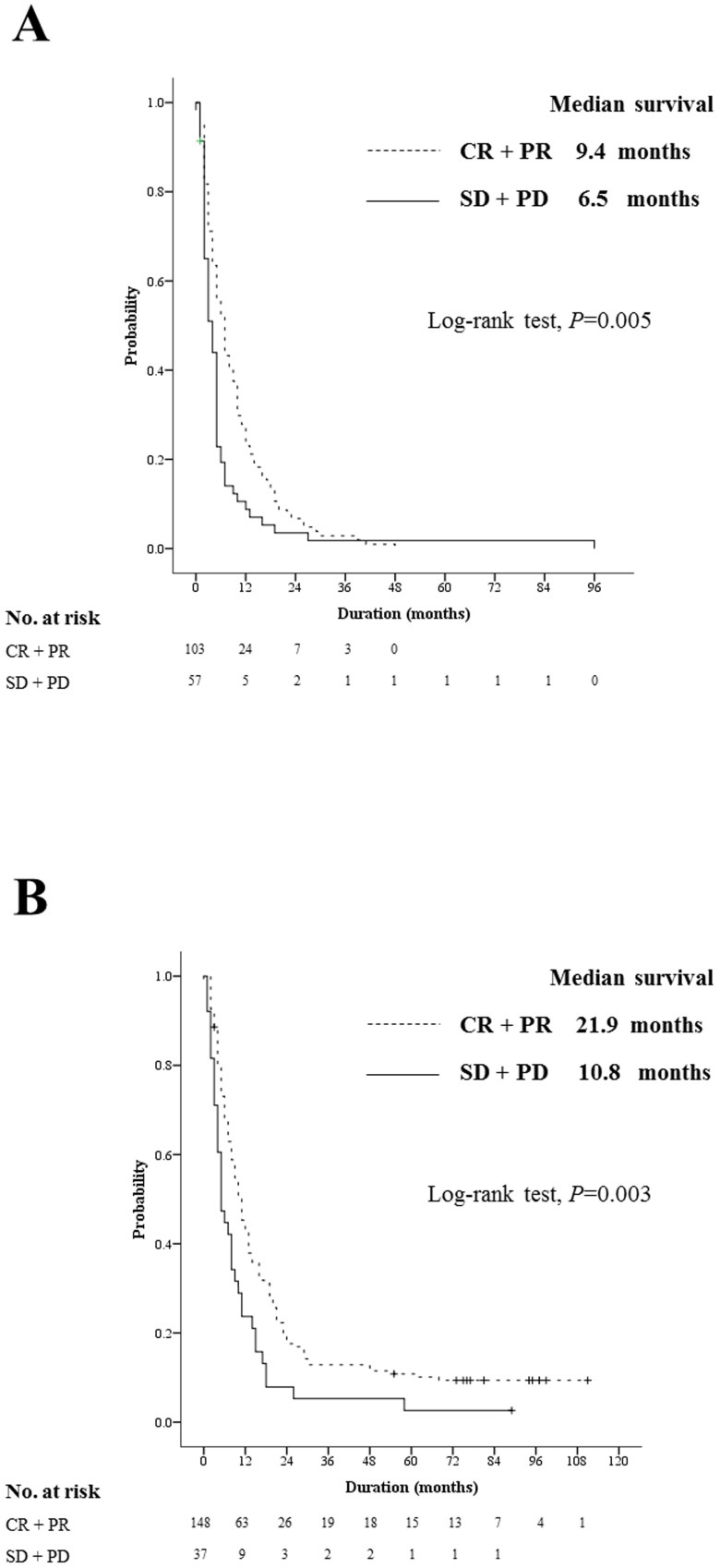
Survival analysis based on tumor response. Responders (CR+PR according to mRECIST, dotted line) showed a better OS, as compared with non-responders (SD+PD, solid line), regardless of the presence of EHM, both (A) in the EHM group (median, 9.4 months vs. 6.5 months; *P* = 0.005) and (B) in the non-EHM group (median, 21.9 months vs. 10.8 months, *P* = 0.003).

The responders to initial TACE demonstrated an improved OS compared with non-responders in both the EHM group (9.4 months vs. 6.5 months; hazard ration [HR], 1.61; 95% confidence interval [CI], 1.15–2.24; *P* = 0.005; Figure 2A) and the non-EHM group (21.9 months vs. 10.8 months; HR, 1.75; 95% CI, 1.21–2.53; *P* = 0.003; Figure 2B).

**Figure 3:**
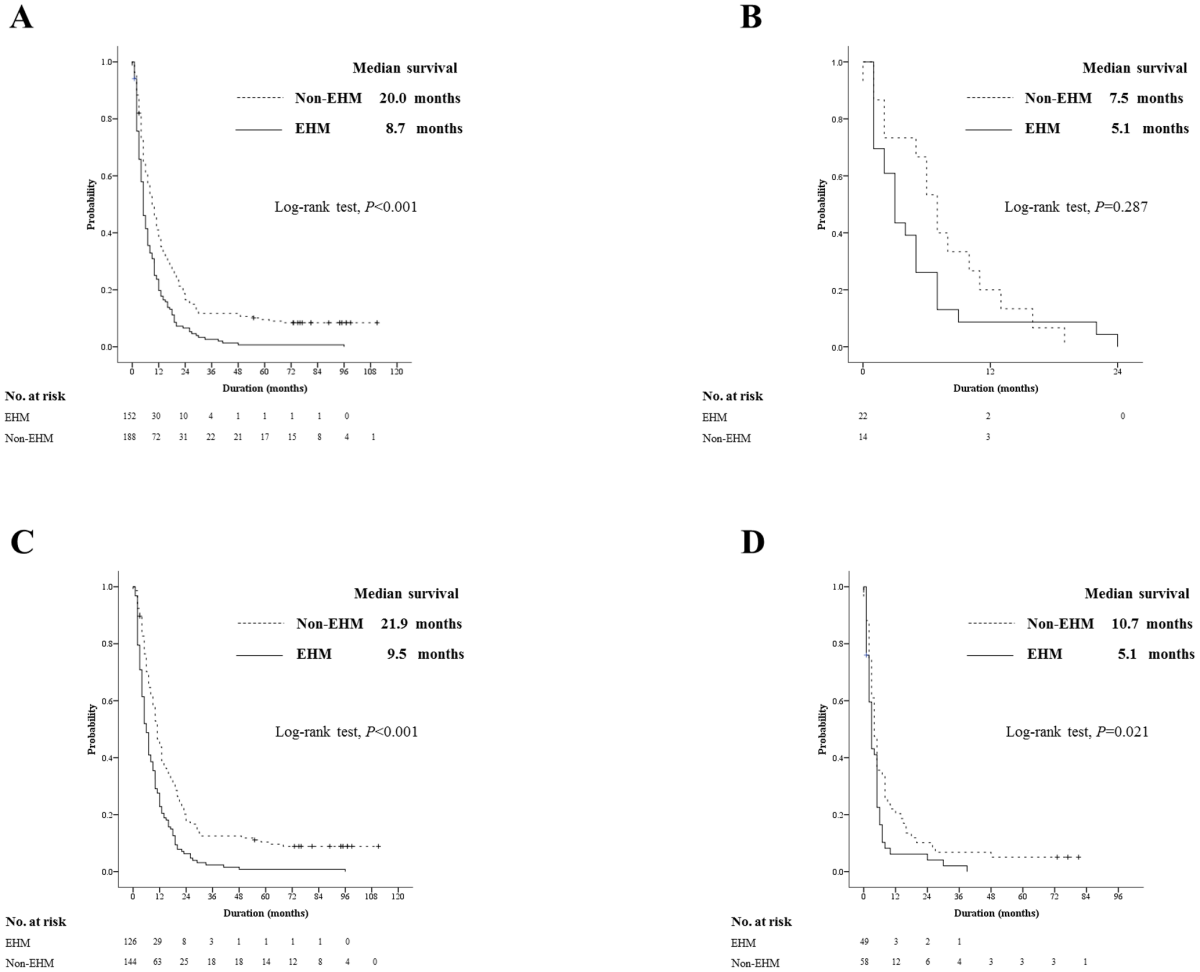
Survival analysis based on intrahepatic stage and liver function. Subgroup analysis based on the intrahepatic stage and liver function was undertaken. The solid line represents patients with EHM and the dotted line for patients without EHM: The graphs demonstrates the comparisons of OS between the EHM group and the non-EHM group (A) in patients with T3 intrahepatic tumor (median, 8.7 months vs. 20.0 months; *P*<0.001), (B) in those with T4 tumor (median, 5.1 months vs. 7.5 months; *P* = 0.287), (C) in those with Child-Pugh class A liver function (median, 9.5 months vs. 21.9 months; *P*<0.001), and (D) in those with Child-Pugh class B (5.1 months vs. 10.7 months; *P* = 0.021), respectively.

As for the subgroup analyses, the EHM group had a significantly shorter OS than the non-EHM group for patients with T3 classifications (median, 8.7 months [range, 1.6–96.0] vs. 20.0 months [range, 4.3–11.0]; HR, 1.71; 95% CI, 1.37–2.13; *P*<0.001; Figure 3A), but not patients with a T4 classification (median, 5.1 months [range, 0.5–24.0] vs. 7.5 months [range, 0.7–19.0]; HR, 1.41; 95% CI, 0.72–2.77; *P* = 0.287; Figure 3B). The OS of the EHM group was significantly shorter than the non-EHM group for patients with not only Child-Pugh class A (median, 9.5 months [range, 0.5–96.0] vs. 21.9 months [range, 0.7–111.0]; HR, 1.85; 95% CI, 1.44–2.38; *P*<0.001; Figure 3C), but also Child-Pugh class B (median, 5.1 months [range, 0.5–39.0] vs. 10.7 months [range, 0.7–81.0]; HR, 1.52; 95% CI, 1.03–2.25; *P* = 0.021; Figure 3D). We also analyzed whether OS was different according to treatment response to EHM. The OS of the responders was not significantly longer than non-responders (13.3 months vs. 11.7 months; HR 1.74; 95% CI, 0.23–2.38; *P* = 0.614).

### Combination therapy with TACE plus systemic therapy versus TACE alone

**Figure 4:**
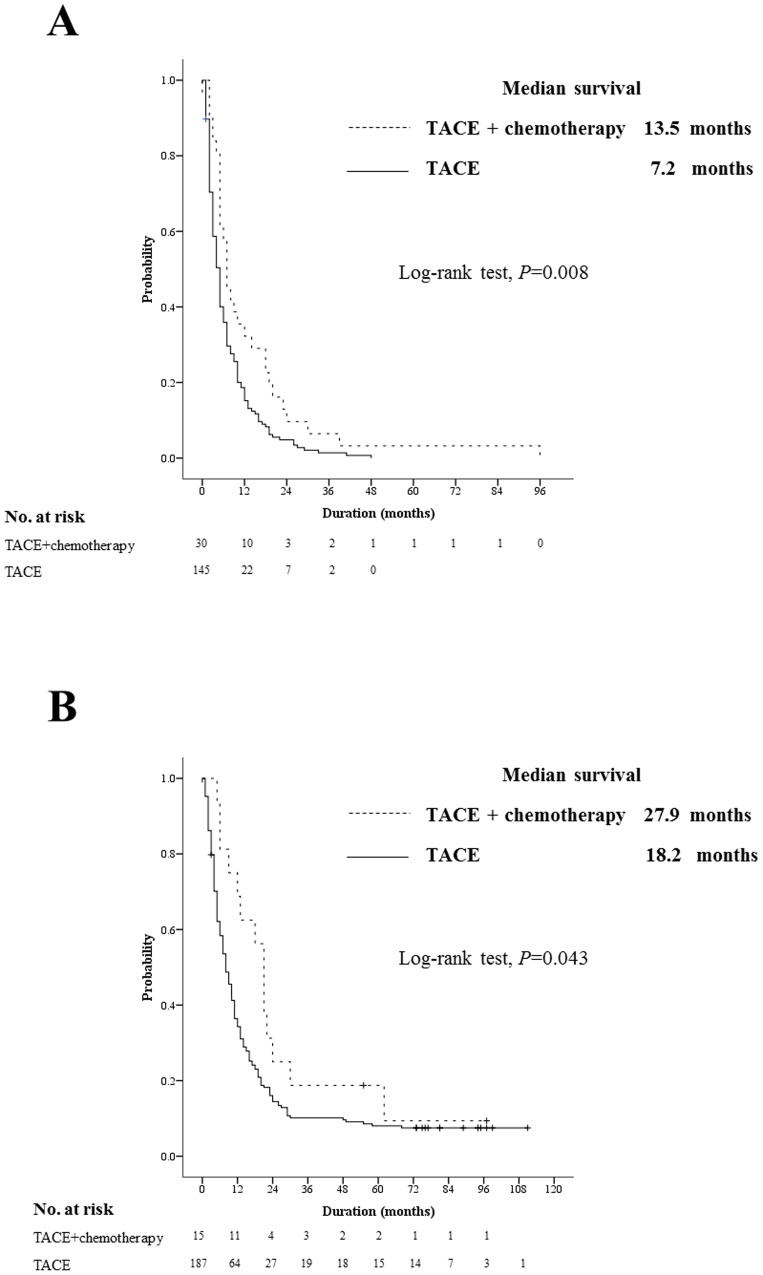
Survival analysis based on treatment modalities. The combination group (TACE with systemic chemotherapy, dotted line) showed a better OS than the TACE group (TACE without systemic chemotherapy, solid line), irrespective of EHM, both (A) in the EHM group (median, 13.5 months vs. 7.2 months; *P* = 0.008) and (B) in the non-EHM group (median, 27.9 months vs. 18.2 months; *P* = 0.043).

Among patients with EHM, 31 patients underwent TACE combined with systemic chemotherapy, while 146 underwent TACE without systemic therapy. A longer OS was demonstrated for patients in the combination therapy group than patients treated with TACE alone in the EHM group (median, 13.5 months vs. 7.2 months; HR, 1.65; 95% CI, 1.11–2.45; *P* = 0.008; Figure 4A). The result was the same for the non-EHM group (median, 27.9 months vs. 18.2 months; HR, 1.71; 95% CI, 1.01–2.96; *P* = 0.043; Figure 4B).

### Cause of death

Eighteen patients (5.0%) were still alive at the end of this study, but 363 (95.0%) had died. The cause of death was investigated for the 174 patients who had died in our hospital (Table 4). In the EHM group, most patients (56, 70.9%) had died because of intrahepatic HCC or liver failure, which was also the most common cause in the non-EHM group (79, 83.2%).

**Table 4:** Cause of death in advanced HCC patients with or without extrahepatic metastases.

Cause of death	EHM (n = 79) Number (%)	Non-EHM (n = 95) Number (%)
**Intrahepatic HCC or liver failure**	56 (70.9)	79 (83.2)
**Extrahepatic HCC**	13 (16.5)	0 (0)
T3	13 (16.5)	
T4	0 (0)	
**Causes not related with liver nor HCC**	10 (12.7)	16 (16.8)

Abbreviations: HCC, hepatocellular carcinoma; EHM, extrahepatic metastases.

Thirteen patients (16.5%) had died because of EHM. Nine patients had died because of respiratory failure related to lung metastases. Two patients had died because of bone metastasis–related disease (e.g., spinal cord compression). Two patients had died because of intracranial hemorrhage secondary to brain metastases. There were no deaths because of lymph node or adrenal gland metastases. All patients who had died of EHM were initially deemed a T3 intrahepatic tumor classification.

## Discussion

The prognosis of patients with advanced HCC is extremely poor. The median survival of untreated patients is about 5.4 months [13]. Sorafenib is the standard first-line treatment for patients with advanced HCC (BCLC stage C), as it is the only systemic therapy proven to prolong OS for these patients. However, sorafenib monotherapy confers approximately 2–3 months of an actual survival gain, as compared with placebo, and its efficacy has not been fully proven in the subgroup of patients with EHM [5], [6]. Therefore, many hepatologists still rely on a conventional ‘liver-first’ approach to treat patients with EHM. In general, TACE is not recommended if EHM is present [7], [14]. However, a recent study showed that repeated TACE treatment could prolong survival for EHM patients who have preserved liver function [15], [16]. Actually, in many centers, TACE is performed for highly selected BCLC stage C patients based on their extent of disease, patient preference, and the presence of symptoms relevant to the organs with EHM [17]. In our study, in which the proportion of BCLC stage C was 100%, advanced HCC patients treated with TACE alone without systemic therapy demonstrated an improved median overall survival of 13.4 months, as compared with previous landmark sorafenib trials, especially in the non-EHM group (18.2 months). Considering that the median survival of the patients administered sorafenib was 10.7 months in the SHARP trial (BCLC stage C, 82%) [5] and 6.5 months in the Asia-Pacific trial (BCLC stage C, 95%) [6], it could be suggested that TACE might be a possible alternative treatment for advanced HCC, particularly for patients with portal vein invasion without EHM.

Our major finding was that patients with advanced HCC (BCLC stage C) do not derive similar benefits from TACE given the presence of EHM. Having the same BCLC stage C does not guarantee a similar overall survival. It means that a BCLC stage C comprises a heterogeneous population, and patients may differ given the presence of EHM. Furthermore, our study demonstrated that the proportion of non-responders was significantly higher in the EHM group than in the non-EHM group. This result showed that the efficacy of TACE for targeting intrahepatic lesions is affected by the presence of EHM, suggesting that the presence of EHM may imply poor tumor biology with rapid tumor progression, resulting in poorer outcomes. Based on the results of recent studies, metastatic HCC may differ from non-metastatic HCC at both the biologic and molecular levels. Several genes including FoxQ1 [18], ACP5 [19], and S100A14 [20] have been recognized as candidate genes for metastasis of HCC. Each gene is known to promote invasion and metastasis of HCC *in vitro* and *in vivo*. From this point of view, BCLC stage C could be subclassified according to the presence of EHM.

Tumor response after the initial TACE treatment correlated well with overall survival for both the EHM and non-EHM groups. And both the EHM group and non-EHM group, most patients died because of intrahepatic HCC and/or following liver dysfunction. It might imply that more effective control of intrahepatic HCC is important to achieve better prognosis. Moreover, the combination therapy group demonstrated a better overall survival than the TACE alone group, and the result was the same regardless of the presence of EHM. It means that not only intrahepatic therapy but also systemic therapy should be preferentially considered for BCLC stage C, irrespective of EHM. Recent phase II trials evaluating concurrent treatment with TACE and sorafenib have provided encouraging results for unresectable HCC [21]. Two small retrospective studies have reported outcomes on combination therapy with TACE plus sorafenib in HCC patients with EHM. One study reported that TACE plus sorafenib was superior to sorafenib alone regarding time to progression for patients with advanced stage HCC (90% had EHM), although it failed to improve OS [15]. Another study reported that in a subgroup analysis, aggressive treatment with TACE in combination with systemic chemotherapy resulted in better outcomes for EHM patients [16].

Our study had a few critical limitations. First, this was a retrospective study with a relatively small size. Second, it included patients who were administered TACE with or without systemic therapy, but not sorafenib alone, a current standard treatment for BCLC stage C patients.

In conclusion, the results of this study show that the prognosis of advanced HCC patients with EHM is significantly worse than that of patients without EHM with repeated TACE treatments with/without systemic therapy, and tumor response was affected by the presence of EHM. The survival rate was higher in the combination therapy group than the TACE alone group. These results indicate that the presence of EHM means aggressive tumor biology and that subclassification of BCLC stage C may be necessary. Further prospective studies are necessary to confirm: (i) whether the presence of EHM could be a major prognostic factor for patients treated with sorafenib, a current standard treatment, and (ii) whether combination therapy with systemic therapy and TACE would further improve the survival of patients with advanced HCC as compared with those treated with either TACE or sorafenib alone.
